# A New Procedure-Based Assessment of Operative Skills in Gastric Bypass Surgery, Evaluated by Video Fragment Rating

**DOI:** 10.1007/s11695-023-07020-4

**Published:** 2024-02-24

**Authors:** Mirjam A. Kaijser, Gabrielle H. van Ramshorst, Bart A. van Wagensveld, Nic J. G. M. Veeger, Jean-Pierre E. N. Pierie, Expert panel of Dutch and Belgian Bariatric Surgeons, Stefan L. Damen, Marc J. van Det, Marloes Emous, Esther D. van den Ende, Ewoud H. Jutte, Eric J. Hazebroek, Gerhard van’t Hof, René A. Klaassen, Barbara S. Langenhoff, Ronald S. L. Liem, Hendrik A. Marsman, Simon W. Nienhuijs, Yves van Nieuwenhove

**Affiliations:** 1 School of Medicine, University of Groningen, University Medical Centre Groningen, Groningen, The Netherlands; 2 Department of Surgery, Medical Centre Leeuwarden, Leeuwarden, The Netherlands; 3 Center for Obesity Northern Netherlands, Medical Center Leeuwarden, Henri Dunantweg 2, 8934 AD Leeuwarden, The Netherlands; 4 Department of Gastrointestinal Surgery, Ghent University Hospital, Ghent, Belgium; 5 Department of Human Structure and Repair, Ghent University, Ghent, Belgium; 6 Weight Management Center, Department of Surgery, NMC Royal Hospital, Khalifa City, Abu Dhabi United Arab Emirates; 7 Department of Epidemiology, Medical Center Leeuwarden, Leeuwarden, The Netherlands; 8 Department of Epidemiology, University of Groningen, University Medical Center Groningen, Groningen, The Netherlands

**Keywords:** LRYGB, Bariatric surgery, Training, Assessment, PBA

## Abstract

**Purpose:**

Feedback on technical and procedural skills is essential during the training of residents and fellows. The aim of this study was to assess the performance of a newly created instrument for the assessment of operative skills using laparoscopic Roux-en-Y gastric bypass (LRYGB) video fragments.

**Materials and Methods:**

A new procedure-based assessment (PBA) was created by combining LRYGB key steps with a 5-point independence scale. LRYGB performed by residents and surgeons with different levels of expertise were video recorded. Fragments of the pouch creation, gastro-jejunostomy and jejunojejunostomy, were review by 12 expert bariatric surgeons and the operative skills assessed with the PBA, *Objective Structured Assessment of Technical Skill* (OSATS), and the Bariatric OSATS (BOSATS). The PBA was compared to the OSATS and BOSATS. Mean scores for all items of the different assessments were summarized and compared using a *T*-test.

**Results:**

The scores of the procedural steps were combined and compared for all levels. The mean scores for beginner, intermediate, and expert level were 2.71, 3.70, and 3.90 for the PBA; for the OSATS 1.84, 2.86, and 3.44; and for the BOSATS 2.78, 3.56, and 4.19. Each of these assessments differentiated between the three skill levels (all *p* < 0.05).

**Conclusion:**

The PBA discriminates well between different levels of operative skills. Similar patterns were found for the OSATS and BOSATS, showing that the randomly selected video fragments are representative samples for assessing skill level. Future research will demonstrate whether these results can be extrapolated to clinical training, and which scores allow for procedure certification.

**Graphical Abstract:**

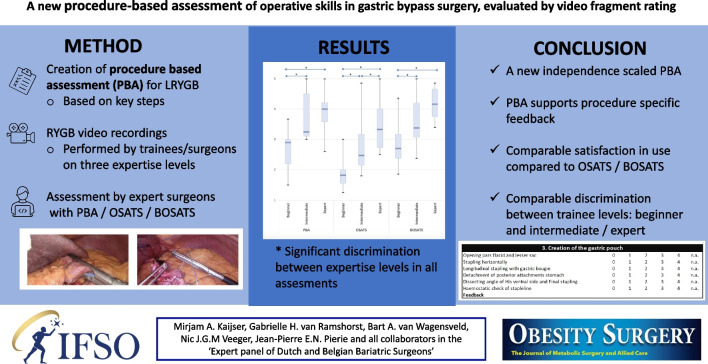

**Supplementary Information:**

The online version contains supplementary material available at 10.1007/s11695-023-07020-4.

## Introduction

Laparoscopic Roux-en-Y gastric bypass is one of the most effective treatments in the pandemic of obesity, resulting in sustainable weight loss, remission of comorbidities, and improved quality of life [1–4]. With the number of procedures still increasing worldwide, training of surgeons who can perform these procedures is essential. LRYGB has a learning curve of around 50–200 procedures, but numbers as high as 500 procedures for complete mastery have been described in literature [5–8]. Several studies have shown that training of residents and fellows during laparoscopic gastric bypass procedure may affect outcomes in terms of complications and costs [9, 10].

In any training situation, assessment is necessary to support feedback in the learning process and, eventually, to prove adequate skill [11, 12]. Residents and other trainees can be given feedback and undergo formal assessments with for example the *Global Rating Scale* (GRS), the *Objective Structured Assessment of Technical Skill* (OSATS), *Global Operative Assessment of Technical Skill* (GOSATS), and *Global Operative Assessment of Laparoscopic Skills* (GOALS) [13–17]. OSATS seems to be the golden standard in surgical training [14–16]. These assessments focus on generic surgical skills, but not on a specific procedure or its crucial steps. Moreover, feedback and assessment by certified surgeons to those who are learning new procedures may be less formal, and the aforementioned assessment may not be validated for these groups [13]. The role of GRS in summative assessments or grading is unsure [16].

When training complex (laparoscopic) procedures such as the LRYGB many surgical resident programs have adopted some sort of stepwise training, in which the different procedural steps are instructed consecutively [18]. Additional operative times, and risks, are kept to a minimum by transferring it to the trainee in only small parts. Likewise, medical training programs are progressing from master-apprentice training to *competency-based medical education* (CBME). Some training programs, including the Dutch Surgical training program, are now based on *entrustable professional activities* (EPAs). In this transition the need for summative feedback next to formative feedback increases [19]. A *procedure-based assessment* (PBA) focuses on the execution of a specific procedure or operation as well as its substeps [20]. PBAs have been created for different surgical procedures, including laparoscopic cholecystectomy, colectomy, fundoplication, and bariatric procedures [21–23]. Several of these are graded in a specific ‘technical performance’ scale specified for each observed item.

Based on previous work of Glarner et al. and Kramp et al. it was aimed to create an *independence scaled* procedure-based assessment on LRYGB and to assure that this assessment can differentiate between different expertise levels of trainees with the use of video recordings [20, 21]. A high level of independence may eventually lead to professional entrustment or certification for this procedure.

## Materials and Methods

First, a new PBA was designed based on a previously introduced PBA in laparoscopic cholecystectomy (LC) [21]. Second, laparoscopic LRYGB procedures performed by residents and surgeons on different levels were video recorded. After selecting specific fragments of these procedures, twelve independent expert bariatric surgeons gave feedback with the PBA, BOSATS, and OSATS. Finally, the experts were invited to give their opinions on the use of the different assessment scales by questionnaire.

### Creation of the Procedure-Based Assessment

In previous research LRYGB was divided into multiple steps and substeps with a hierarchical task analysis. A Delphi consensus analysis among Dutch bariatric surgeons defined the key steps of the procedure: the steps that are necessary to safely accomplish the procedure. Based on this Delphi consensus the LRYGB was divided in 9 steps and 44 advised or crucial substeps [24]. Following Kramp et al. in their research on a PBA in LC, these 9 steps were combined into 6 key steps and 30 substeps to improve usability of the PBA [21]. The different items were combined with a 5-point operative independence scale introduced by Glarner [20]. The complete PBA is shown in Supplementary material A.

### Sample Size Calculation

As this study aims to validate a new assessment the sample size calculation was based on comparable research in the existing literature. In a study validating a PBA for LC, which also used one video per expertise level, a relevant difference between levels was found using 10 experts as assessors [21]. As LRYGB was expected to be of higher complexity level than LC, it was assumed that the mutual differences were larger. No data were available to state this difference in a sample size number. In other research Zevin et al. calculated a minimum inclusion of 8 assessments in a comparable study on LRYGB [23].

For this they used the GOALS study of Vassilou et al. to support a relevant difference between novice and expert of 6.4, with a standard deviation of 4.5 and combined this with the power set at 0.8 and an alpha of 0.05 [17]. As three expertise levels and three procedural steps were included, this study involved 9 assessment. Twelve experts were identified as assessors to ensure the expected sample size of 8–10 was reached.

### Video Recordings

Video recordings from LRYGB performed by residents and surgeons were selected from a high-volume bariatric center in the Netherlands. In bariatric procedures a Endoeye HD II camera (© 2020 Olympus America) is used. Recordings are made and stored with the IBoxTouchCompact system (© meso international GmbH).

Two residents were selected, a beginner who had performed < 10 LRYGB and an intermediate experienced resident who had participated in 10–100 procedures as first surgeon. Moreover, one bariatric surgeon with 10 years of experience was selected to include an expert level procedure. All agreed to participate and gave written consent. The OR logs were reviewed and the most recent cases meeting the inclusion criteria were included: all standard bariatric cases, i.e., female subjects with a BMI < 45 without previous abdominal surgery.

After explicit and written consent of the patients to use the anonymous recording of the performed procedure for research and educational purposes, these recordings were downloaded and edited before publication on the study website.

### Video Editing

Video recordings were edited with IMovie version 10.2.1 (© 2001–2020 Apple Inc.). The recorded cases show a LRYGB performed with a linear stapling technique of both the jejunojejunostomy and the antecolic antegastric gastro-jejunal anastomosis. In this study only the PBA fragments of entirely laparoscopic and crucial steps were selected: the creation of the pouch, the biliopancreatic limb (BPL) and gastro-jejunostomy (GJ), and third the alimentary limb (AL) and jejuno-jejunostomy (JJ). Fragments were selected starting from the first grasp and retraction of stomach and lesser omentum to start the pouch, up to retraction of the camera after completing the pouch in the angle of His. For the BPL and GJ fragment recording was started at the first attempt of grasping the pouch to open it for the stapler — up to the completion of the anastomosis by cutting the last stitch. For the alimentary limb and JJ the clip was started from the opening of the first limb, again ending with cutting the last stitch. Each fragment was edited to enhance visibility with a standardized stepwise manner:

- [1] Cut out all instrument changes and instruction moments,
- [2] Speed up specific fragments (firing stapling 15 s, running small bowel),
- [3] Add text information about these changes, as well as supervisor take overs, and
- [4] Add an instruction screen of 20 s.

### Expert Assessor Panel

Twelve experienced bariatric surgeons were included in a new Expert Panel of Dutch and Belgian Bariatric Surgeons. Of each Dutch hospital performing over 700 LRYGB a year one bariatric surgeon was invited to participate. The other half of selected experts were invited based on their specific interest in (bariatric) surgical training. In a randomized order each assessor viewed a fragment of the pouch, creation of BPL and GJ and creation of AL and JJ, each on a different operative experience level. Randomization was done with a Graeco-Latin Square design (PASS-11) [25].

### Comparable Assessments

Two different assessments were selected to evaluate the potential of this new PBA. First, the Dutch golden standard for assessment and feedback in resident training, the OSATS, was included. Second, the BOSATS created by Zevin et al. was used as a comparison [23]. The BOSATS is an example of a PBA with specified scoring criteria for each observed item. As the BOSATS and PBA both use a 5-point Likert scale a version of the OSATS with a 5-point scale was used. An overview of the OSATS and the relevant items of the BOSATS are shown in Supplementary materials B and C. A Dutch of the OSATS translation was derived from Strating et al. and is available at pbassurgery.com [14, 26].

The presented PBA has 30 items in total, divided over 6 steps, of which 16 items (3 steps) were used in this study. Assuming a direct trocar introduction and above-mentioned technique the BOSATS has 64 items in total of which 34 were relevant to the specific steps in this study. The OSATS has 7 items; as it is a global rating scale, we used the full OSATS on all steps. This is summarized in Table 1. A full list of the observed items is found in Supplementary materials A, B, and C.

**Table 1:** Number of observed items in all assessments

	PBA no. of items	BOSATS no. of items	OSATS no. of items	Total no. of items
Pouch creation	6	14	7	27
BPL and GJ	6	13	7	26
AL and JJ	4	7	7	18

*BPL* biliopancreatic limb, *GJ* gastro-jejunal anastomosis, *AL* alimentary limb, *JJ* jejunojejunostomy

### Website

To facilitate viewing the videos online a web-based application was created at https://pbasurgery.com, available during the study period in December 2021. For this specific study a password protected log-in page was used. Personalized log-in details were sent by e-mail to the assessors. After logon, the assessor was directed to the main screen (Supplementary material D) with entry to the different videos and surveys. Clicking a video opened a next screen showing the video followed by the relevant items of the BOSATS and PBA, as well as an OSATS were presented to the viewer. Participants were allowed to pause, fast-forward, or lookback in videos. To make the assessments comparable regarding face of the content all responses on all items were presented in a drop-down menu. Only after entering all responses assessors could use the send button, after which the data were sent to a cloud-based password-protected database and the participant redirected to the main screen. Review or change of answers afterwards was not possible. This assured an anonymous transfer of the data to the researchers, while allowing reminders to be sent after 2 weeks to assessors who had not completed all assessments. Reminders were sent after 2 and 4 weeks, after which all assessors had completed the assigned tasks in full.

After assessing all three fragments the experts responded to two questionnaires, one about their bariatric experience and one regarding the usability of the different assessments. This last questionnaire was re-used from the research of Kramp et al. in Supplementary material E [21].

### Statistical Analysis

The scores on PBA, OSATS, and BOSATS are presented using the mean and standard deviation or median and IQR, depending on normality of data. The data of each assessment are presented both overall and per surgical step (i.e., pouch creation, BPL and GJ, and AL and JJ). The group comparisons of the ratings of the recorded surgical procedures on the beginner, intermediate, and expert level were performed using one-way univariate ANOVA (*F* test) for normally distributed variables or Kruskal–Wallis test for skewed distributed variables. For additional two-group comparisons a Student-*T*- or Mann Whitney *U* test was used.

A 2-tailed *p*-value less than 0.05 indicates statistical significance. In the additional secondary two-group tests no correction for multiple testing was applied. All analyses were performed using SAS version 9.4.

The results of the questionnaires were presented with descriptive statistics.

## Results

### Patient Characteristics

For the intermediate-level resident, as well as the surgeon, videorecorded procedures including all operative steps were included. For the beginner the recorded steps were divided among two patients — the supervising attending surgeon performed the other steps in these operations. Characteristics of the patients recorded in the included videos are summarized in Table 2.

**Table 2:** Characteristics of the recorded cases

Patient	Gender	Age	BMI	Comorbidities	Surgeon expertise	Recorded step
1	V	54	36	DM, HT, OSAS	Beginner	Pouch creation
1	V	54	36	DM, HT, OSAS	Beginner	AL and JJ
2	V	51	42	HT	Beginner	BPL and GJ
3	V	56	43	Arthrosis	Intermediate	All steps
4	V	52	42	–	Expert	All steps

*BMI* body mass index, *BPL* biliopancreatic limb, *GJ* gastro-jejunal anastomosis, *AL* alimentary limb, *JJ* jejunojejunostomy, *DM* diabetes mellitus, *HT* hypertension, *OSAS* obstructive sleep apnea syndrome

### Surgical Staff

The included surgical staff were a PGY 2 resident with < 100 case laparoscopic experience and less than 10 LRYGB cases, a PGY 6 resident (the Dutch Surgical training program has a 6-year curriculum) with 100–500 laparoscopic cases as first surgeon, but less than 100 LRYGB cases and finally an attending surgeon with over 1000 laparoscopic cases and > 500 LRYGB cases were selected (Table 3).

**Table 3:** Characteristics of the (resident) surgeons

Subject	Expertise level	PGY	Laparoscopic cases	LRYGB cases
1	Beginner	2	10–100	< 10
2	Intermediate	6	100–500	10–100
3	Expert	Surgeon	> 1000	> 500

*PGY* post-graduate year, *LRYGB* laparoscopic Roux-and-Y gastric bypass

### The Expert Assessor Panel

The expert team consisted of three women and 9 males. All assessors had performed over 1000 laparoscopic cases. Two experts had performed 500–1000 bariatric cases, all others > 1000 cases. Ten experts had over 10 years of experience after training. Two attending surgeons had 5–10 years of clinical experience; both had > 1000 laparoscopic cases and > 500 LRYGB procedures.

### The Assessments

**Fig. 1:**
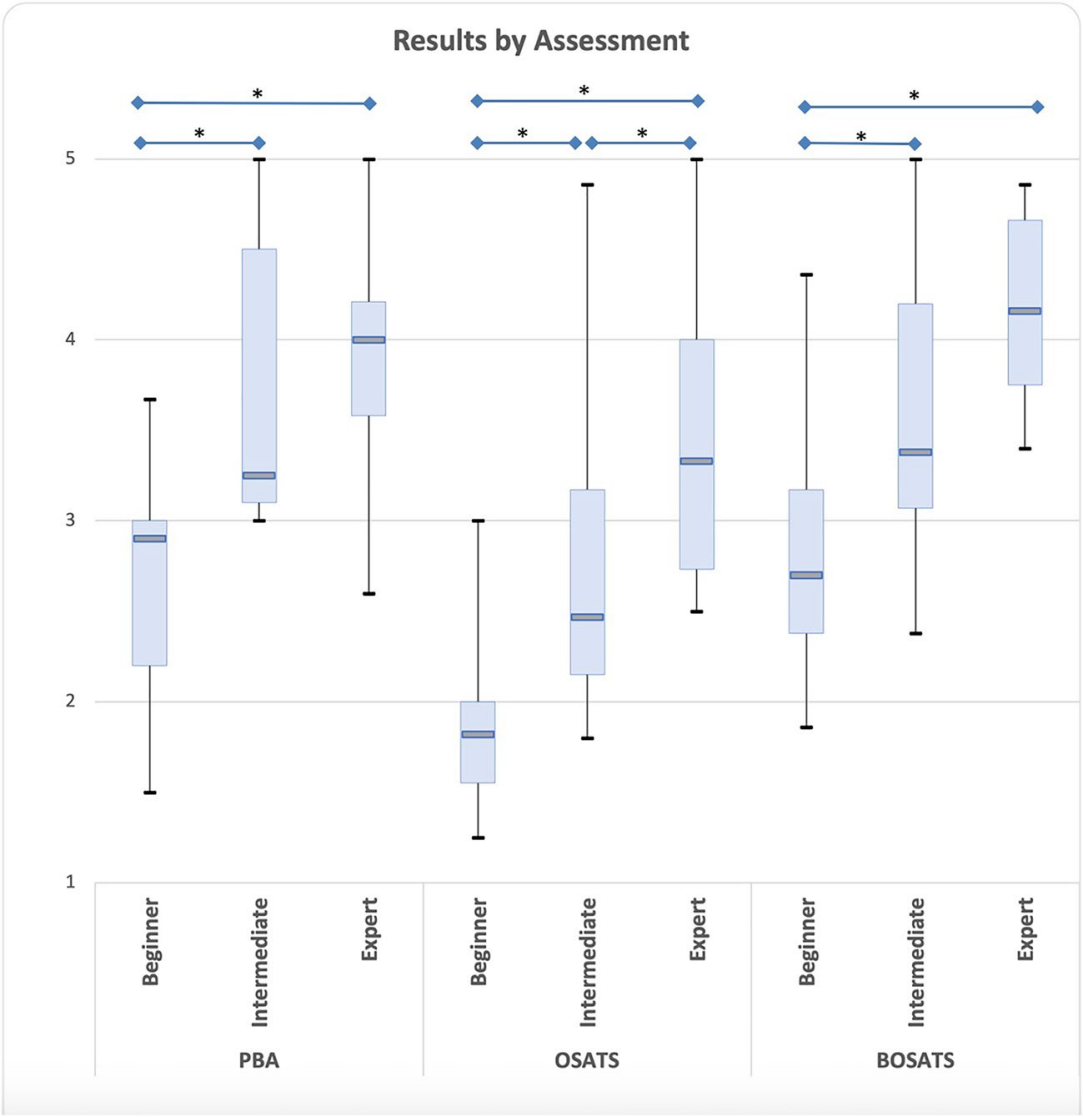
Median score, interquartile range (IQR), and minimum and maximum scores of the aggregate of substeps in the procedure-based assessment (PBA), Objective Structured Assessment of Technical Skill (OSATS), and Bariatric OSATS (BOSATS) on three levels. An asterisk indicates a significant difference

The mean scores of the items in the substeps and standard deviation (SD) are shown in Table 4. In all assessments the overall scores increased with the level of experience. However, in the *BPL and GJ* steps the intermediate surgeon gained higher mean scores than the expert (4.75 vs 4.00, *p* = 0.005). As this was not the case in the other steps (all *p* > 0.05), *creation of the gastric pouch* and *AL and JJ*, taking all steps together the three assessments could differentiate between the different expertise levels. However, for the *BPL and GJ* the BOSATS was not significant. The same was found in the *creation of the gastric pouch* in the PBA. Figure 1 shows the median scores, interquartile range, and minimum and maximum scores on these assessments.

**Table 4:** Mean score and standard deviation (SD) of the three observed substeps and the aggregate of all these steps

	Beginner		Intermediate		Expert		*P*-value			
	Mean	SD	Mean	SD	Mean	SD	Overall	B vs I	B vs E	I vs E
PBA										
All steps	2.71	0.64	3.70	0.81	3.90	0.67	< 0.001*	0.003*	< 0.001*	0.52
Pouch creation	2.80	0.28	3.13	0.14	3.19	0.50	0.28	0.087	0.22	0.82
BPL and GJ	3.21	0.46	4.75	0.32	4.00	0.14	< 0.001*	0.002*	0.016*	0.005*
AL and JJ	2.13	0.63	3.21	0.17	4.50	0.46	< 0.001*	0.016*	< 0.001*	0.002*
BOSATS										
All steps	2.78	0.72	3.56	0.82	4.19	0.50	< 0.001*	0.022*	< 0.001*	0.032*
Pouch creation	2.63	0.22	3.04	0.51	4.16	0.34	< 0.001*	0.19	< 0.001*	0.010*
BPL and GJ	3.56	0.61	4.45	0.41	3.87	0.64	0.13	0.052	0.51	0.18
AL and JJ	2.14	0.33	3.18	0.64	4.54	0.32	< 0.001*	0.029*	< 0.001*	0.009*
OSATS										
All steps	1.84	0.47	2.86	0.99	3.44	0.80	< 0.001*	0.004*	< 0.001*	0.13
Pouch creation	1.78	0.31	2.24	0.11	3.48	0.61	< 0.001*	0.033*	0.003*	0.007*
BPL and GJ	2.20	0.54	3.96	0.93	2.79	0.25	0.011*	0.017*	0.095	0.052
AL and JJ	1.55	0.35	2.39	0.52	4.05	0.93	0.001*	0.037*	0.002*	0.021*

*B* beginner, *I* intermediate, *E* expert, *BPL* biliopancreatic limb, *GJ* gastro-jejunal anastomosis, *AL* alimentary limb, *JJ* jejunojejunostomy

^*^Significant values (*p* < 0.05)

The OSATS, although significant discriminating between all steps overall, could not make a significant distinction between the two highest proficiency levels, intermediate and expert, neither for the total of steps nor for all separate steps.

### Assessment Preferences

**Fig. 2:**
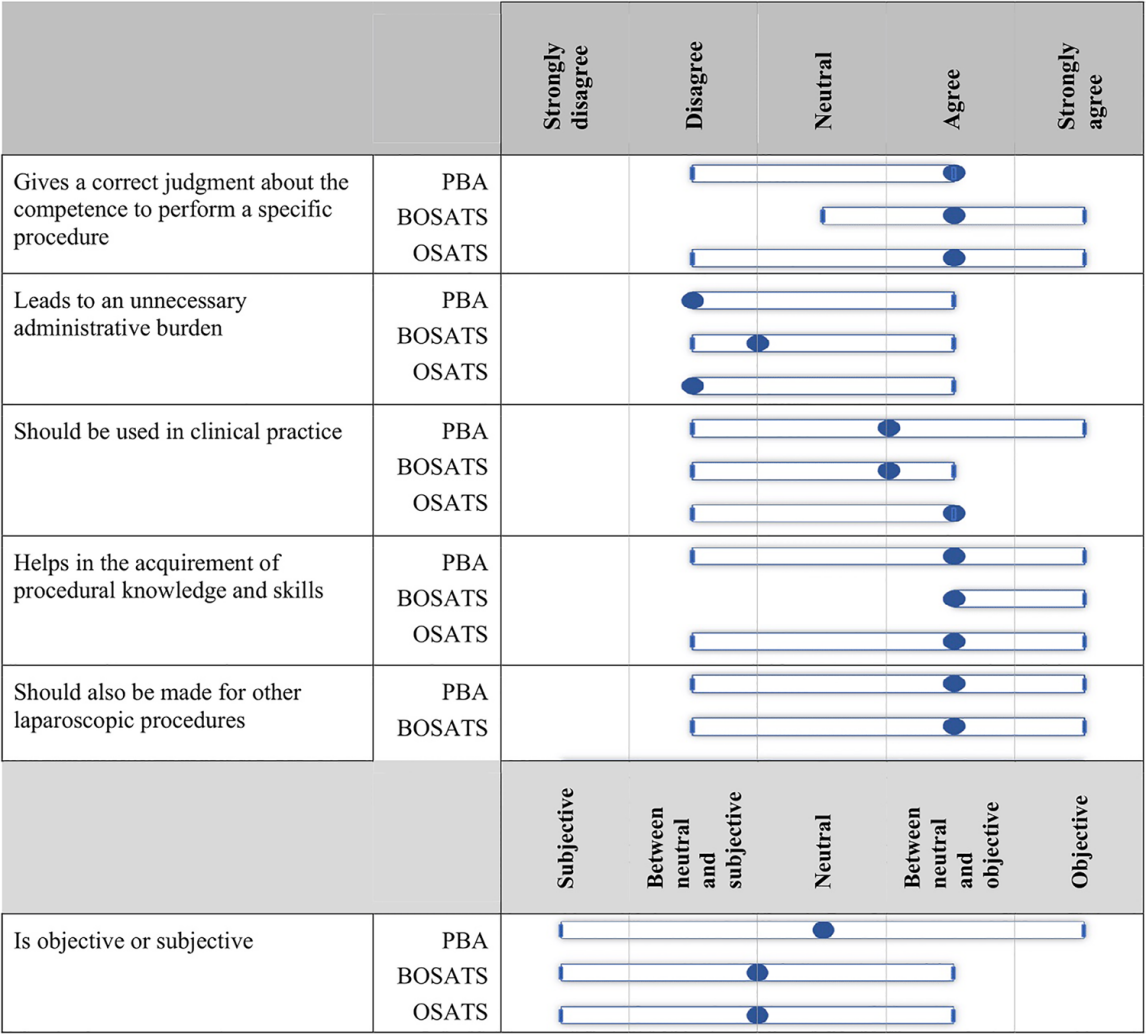
Median scores and range of the assessment preferences

In Fig. 2, the median and range of the scores on the six questions regarding the preferences of the assessors regarding the use of the three assessments are shown. In general, the assessors rated all three assessments alike; on all questions, the median scores did not differ more than 0.5 points.

With a median score of 4 more than half of the assessors agreed that the three assessments give a procedure-specific competency level and (with a median of 2–2.5) do not lead to an unnecessary administrative burden.

More than half of the expert panel (7/12) (strongly) agreed that the PBA and BOSATS (or a similar assessment) should be available for other procedures. Half of the expert panel agreed that the PBA and BOSATS should be used in clinical practice, as the OSATS already is. The BOSATS was considered most helpful in the acquirement of procedural knowledge and skills.

## Discussion

This study introduced a new created procedure-based assessment (PBA) for LRYGB using an independence scale combined with the key steps of the procedure. It was observed that the assessor can use the PBA on a videorecorded LRYGB procedure performed by a novice, intermediate, and expert surgeon to differentiate their competency level. Thus, the PBA provides a relevant assessment of the trainee’s skills. This PBA sets apart from other procedure-based assessments, as the BOSATS, in the use of an independence scale instead of a specified level for each substep. This may make the instrument easier to read as well as more robust in slightly changing operative techniques. Moreover, by using only 30 predefined key steps for the whole procedure this may enhance usability and shorten the duration of the assessment.

Compared to the OSATS, a global rating scale assessment, both this PBA LRYGB and the Bariatric OSATS (BOSATS) show a smaller difference in scores on one specific substep — respectively *the creation of the gastric pouch* and *the biliopancreatic limb and gastro-jejunal anastomosis*. However, if all videorecorded are steps combined, all three instruments can differentiate between the three proficiency levels*.*

Implementing a procedure-based assessment can enhance both summative and formative feedback during surgical training [27]. GRSs are valid instruments during training; however, their construct validity at senior training levels is debatable [13]. For the OSATS scores in this study the same pattern was seen, as the OSATS was less able to discriminate between intermediate and expert level. For advanced trainees such as fellows or attending surgeons, a PBA may be able to provide valuable feedback for learning specific procedures. For beginners, who can perform just a part of the procedure, PBAs can be used to give feedback on that specific step. Moreover, a key step-based assessment can support the technical discussions of the procedures between trainees and supervisors.

Although PBAs have been constructed for different procedures including the BOSATS for LRYGB, no PBA has been implemented in the Dutch Surgical curriculum up to now. This may be explained by the broad spectrum of surgical variations in LRYGB. Earlier studies have shown that a linear stapling technique of both the jejunojejunostomy and the antecolic antegastric gastro-jejunal anastomosis is the standard method of operation in the Netherlands [24]. This new PBA LRYGB follows this technique. Based on a previous Delphi consensus, the LRYGB was divided in 9 steps and 44 advised or crucial substeps. Following Kramp et al. in research on a PBA in LC these 9 steps were combined into 6 steps and 30 substeps to improve usability [21]. Another difference with the BOSATS is the use of a 5-point Likert scale based on *independence.* Most procedural assessments specify the requirements for a specific score for a specific item. This may lengthen the time needed to complete the assessment.

A remarkable result is that the surgeon with intermediate experience had a higher PBA score than the expert on the biliopancreatic limb and gastro-jejunal anastomosis. Looking at the free text feedback of the assessor panel, although the steps were based on a Delphi consensus, they commented to execute this step with a slightly different technique than performed by the expert surgeon, which might have influenced their judgment [24]. In clinical practice this would be less relevant assuming the trainee would, in general, follow their supervising attending technique.

A key difference between the current and previous PBA studies is that a third proficiency level was included [21, 28]. With the learning curve of the LRYGB still not fully defined the choice of the intermediate level between 10 and 100 procedures might be too broad a range.

A limitation of this study is that it only addressed a part of the PBA LRYGB. In this study only the full laparoscopic steps were used as only video recordings of the laparoscopic camera were available. Future research with this PBA LRYGB should include all steps. The use of video recordings in an assessment was validated in previous research [29, 30]. The recordings were shortened in a stepwise manner to ensure raters would not be influenced by the duration of the video, either biased after guessing the proficiency level or reviewer fatigue. The fragments did not include sounds, when relevant a supervisor take-over was displayed in text. Another limitation is that all video recordings showed a standard LRYGB case of females with a BMI < 45 and no previous abdominal surgery to make the video recordings comparable. Although other studies in assessment and training have used similar patients [31], further research is needed to conclude that the assessment is also feasible in more complex cases. As next to the LRYGB the most widely used bariatric procedure in the Netherlands is the gastric sleeve resection, both procedures are a part of the Entrustable Professional Activities (EPAs) in the Dutch Surgical curriculum. A PBA for the gastric sleeve resection will be created in future research.

Although this study focuses on the technical aspects and training of the LRYGB, the training of residents and fellows should include knowledge of the pathophysiological aspects of obesity, care pathways, and treatment plans in metabolic and bariatric surgery. These non-technical skills are a considerable part of the Dutch Surgical curriculum.

## Conclusion

In conclusion, the procedure-based assessment LRYGB is a novel tool in the arsenal of formative and summative assessment in surgical training. As this PBA LRYGB uses an independence scale instead of specified scoring criteria for each step, it is easily readable and has an administrative load comparable to the OSATS. This study shows that this assessment can differentiate between three proficiency levels, taking three videorecorded steps into account. In using predefined key steps and proficiency levels it may be easier to use and less technique depended. We propose to use a PBA as an add on to the use of global rating scales. While many assessments are valid for formative feedback, only a few have shown to be valid in summative scoring or credentialing. Further research should show if this new PBA LRYGB is valid in summative feedback.

### Supplementary Information

Below is the link to the electronic supplementary material.

Supplementary file1 (DOCX 20 KB)

Supplementary file2 (DOCX 34.7 KB)

Supplementary file3 (DOCX 14.2 KB)

Supplementary file4 (DOCX 41.7 KB)

Supplementary file5 (DOCX 1.34 MB)

Supplementary file6 (DOCX 14.2 KB)

## Data Availability

Data sets generated during the current study are available from the corresponding author on reasonable request.
