# Supplementary material for: A New Procedure-Based Assessment of Operative Skills in Gastric Bypass Surgery, Evaluated by Video Fragment Rating
Source: Obes Surg. 2024 Feb 24;34(4):1113–21. doi: 10.1007/s11695-023-07020-4 (PMC11026254; doi:10.1007/s11695-023-07020-4)
Supplement: Supplementary file 1 — Supplementary file1 (DOCX 20 KB) [file 11695_2023_7020_MOESM1_ESM.docx]

## **Supplement A - Video editing**

A four-step editing paradigm was used to make the videos as comparable as possible.

[1] cut out all instrument changes and instruction moments,

[2] speed up specific fragments (firing stapling 15 seconds, running small bowel)

[3] add text information about these changes, as well as supervisor take overs.

[4] add an instruction screen of 20 seconds.

| **Level** | **Step** | **Unedited (mm:ss)** | **Edited (mm:ss)** | **Reduction %** |
| --- | --- | --- | --- | --- |
| Beginner | Pouch | 29:29 | 17:30 | 41,8% |
| Beginner | BPL&GJ | 50:53 | 24:31 | 52,5% |
| Beginner | AL&JJ | 33:25 | 20:07 | 40,8% |
| Intermediate | Pouch | 18:14 | 14:02 | 24,9% |
| Intermediate | BPL&GJ | 18:52 | 14:52 | 23,0% |
| Intermediate | AL&JJ | 26:44 | 16:33 | 39,3% |
| Expert | Pouch | 09:13 | 07:08 | 26,2% |
| Expert | BPL&GJ | 10:18 | 09:14 | 13,6% |
| Expert | AL&JJ | 07:10 | 07:13 | 4,0% |
|  |  |  |  |  |
| *Beginner* | *All* | *01:53:47* | *01:02:08* | *46,3%* |
| *Intermediate* | *All* | *01:03:50* | *00:45:27* | *30,4%* |
| *Expert* | *All* | *00:26:41* | *00:23:35* | *15,4%* |

##### **Table** – Effects of video editing on time

*BPL Biliopancreatic Limb, GJ gastro-jejunal anastomosis, AL alimentary limb, JJ jejunojejunostomy*
