# Supplementary material for: A New Procedure-Based Assessment of Operative Skills in Gastric Bypass Surgery, Evaluated by Video Fragment Rating
Source: Obes Surg. 2024 Feb 24;34(4):1113–21. doi: 10.1007/s11695-023-07020-4 (PMC11026254; doi:10.1007/s11695-023-07020-4)
Supplement: Supplementary file 2 — Supplementary file2 (DOCX 34.7 KB) [file 11695_2023_7020_MOESM2_ESM.docx]

## **Appendix A – PBA LRYGB**

The Dutch version is available at pbasurgery.com

| **Procedure assessment - Laparoscopic LRYGB** | | | | | | |
| --- | --- | --- | --- | --- | --- | --- |
|  |  |  |  |  |  |  |
| **Rating scale** |  |  |  |  |  |  |
| Did not perform this step | 0 |  |  |  |  |  |
| Performs some part of the task, needs assistance to complete task | 1 |  |  |  |  |  |
| Performs the task with direct guidance & instruction | 2 |  |  |  |  |  |
| Performs the task with minimal guidance & instruction | 3 |  |  |  |  |  |
| Performs the task with no guidance or instruction | 4 |  |  |  |  |  |
| Not applicable | na. |  |  |  |  |  |
|  |  |  |  |  |  |  |
| The order of these steps is not relevant for scoring | | | | | | |
|  |  |  |  |  |  |  |
| **1. Operative set-up** | | | | | | |
| Positioning of monitors and OR team | 0 | 1 | 2 | 3 | 4 | na |
| Positioning patient | 0 | 1 | 2 | 3 | 4 | na |
| Time-out procedure including checking antibiotic prophylaxis | 0 | 1 | 2 | 3 | 4 | na |
| Disinfection, sterile exposure | 0 | 1 | 2 | 3 | 4 | na |
| **Feedback** |  |  |  |  |  |  |
|  |  |  |  |  |  |  |
| **2. Starting the laparoscopy** | | | | | | |
| Introduction optical trocart | 0 | 1 | 2 | 3 | 4 | na |
| Introduction of additional trocars under laparoscopic sight | 0 | 1 | 2 | 3 | 4 | na |
| Introduction of liver retractor | 0 | 1 | 2 | 3 | 4 | na |
| Exposure of operative field | 0 | 1 | 2 | 3 | 4 | na |
| **Feedback** |  |  |  |  |  |  |
|  |  |  |  |  |  |  |
| **3. Creation of the gastric pouch** | | | | | | |
| Opening pars flacida and lesser sac | 0 | 1 | 2 | 3 | 4 | na |
| Stapling horizontally | 0 | 1 | 2 | 3 | 4 | na |
| Longitudinal stapling with gastric bougie | 0 | 1 | 2 | 3 | 4 | na |
| Detachment of posterior attachments stomach | 0 | 1 | 2 | 3 | 4 | na |
| Dissecting angle of His ventral side and Final stapling | 0 | 1 | 2 | 3 | 4 | na |
| Haemostatic check of stapleline | 0 | 1 | 2 | 3 | 4 | na |
| **Feedback** |  |  |  |  |  |  |
|  |  |  |  |  |  |  |
| **4. Biliopancreatic limb and gastro-jejunal anastomosis** | | | | | | |
| Lift transverse colon and Identification of Treitz' ligament | 0 | 1 | 2 | 3 | 4 | na |
| Measure jejunum starting from Treitz' ligament | 0 | 1 | 2 | 3 | 4 | na |
| Checking possibility for a tension free anastomosis | 0 | 1 | 2 | 3 | 4 | na |
| Opening of the pouch and jejunum | 0 | 1 | 2 | 3 | 4 | na |
| Stapled gastro-jejunal anastomosis | 0 | 1 | 2 | 3 | 4 | na |
| Completing gastro-jejunal anastomosis with sutures | 0 | 1 | 2 | 3 | 4 | na |
| **Feedback** |  |  |  |  |  |  |
|  |  |  |  |  |  |  |
| **5. Alimentary limb and entero-enteral anastomosis** | | | | | | |
| Determine length of alimentary limb | 0 | 1 | 2 | 3 | 4 | na |
| Opening efferent and afferent limb | 0 | 1 | 2 | 3 | 4 | na |
| Stapled entero-enteral anastomosis | 0 | 1 | 2 | 3 | 4 | na |
| Completing entero-enteral anastomosis with sutures | 0 | 1 | 2 | 3 | 4 | na |
| **Feedback** |  |  |  |  |  |  |
|  |  |  |  |  |  |  |
| **6. Finishing bypass en operation** | | | | | | |
| Leaktest | 0 | 1 | 2 | 3 | 4 | na |
| Transecting small bowel between gastro-jejunal and entero-enteral anastomosis | 0 | 1 | 2 | 3 | 4 | na |
| Closure of mesenteric defects | 0 | 1 | 2 | 3 | 4 | na |
| Removal of liver retractor | 0 | 1 | 2 | 3 | 4 | na |
| Removal of trocarts | 0 | 1 | 2 | 3 | 4 | na |
| Sign out | 0 | 1 | 2 | 3 | 4 | na |
| **Feedback** |  |  |  |  |  |  |
