# Supplementary material for: A New Procedure-Based Assessment of Operative Skills in Gastric Bypass Surgery, Evaluated by Video Fragment Rating
Source: Obes Surg. 2024 Feb 24;34(4):1113–21. doi: 10.1007/s11695-023-07020-4 (PMC11026254; doi:10.1007/s11695-023-07020-4)
Supplement: Supplementary file 3 — Supplementary file3 (DOCX 14.2 KB) [file 11695_2023_7020_MOESM3_ESM.docx]

## Appendix B - OSATS

| Please circle the number corresponding to the candidate’s performance in each category, irrespective of training level | | | | | |
| --- | --- | --- | --- | --- | --- |
| **Respect for tissue:** | 1 Frequently used unnecessary force on tissue or caused damage by inappropriate use of instruments | 2 | 3 Careful handling of tissue but occasionally caused inadvertent damage | 4 | 5 Consistently handled tissues appropriately with minimal damage |
| **Time and motion:** | 1 Many unnecessary moves | 2 | 3 Efficient time/motion but some unnecessary moves | 4 | 5 Clear economy of movement and maximum efficiency |
| **Knowledge and handling of instrument:** | 1 Lack of knowledge of instruments | 2 | 3 Competent use of instruments but occasionally appeared stiff or awkward | 4 | 5 Obvious familiarity with instruments |
| **Flow of operation:** | 1 Frequently stopped procedure and seemed unsure of next move | 2 | 3 Demonstrated some forward planning with reasonable progression of procedure | 4 | 5 Obviously planned course of procedure with effortless flow from one movement to the next |
| **Use of assistants:** | 1 Consistently placed assistants poorly or failed to use assistants | 2 | 3 Appropriate use of assistants most of the time | 4 | 5 Strategically used assistants to the best advantage at all times |
| **Knowledge of specific procedure:** | 1 Deficient knowledge. Needed specific instructions at most steps | 2 | 3 Knew all important steps of procedure | 4 | 5 Demonstrated familiarity with all aspects of operation |
