# Supplementary material for: A New Procedure-Based Assessment of Operative Skills in Gastric Bypass Surgery, Evaluated by Video Fragment Rating
Source: Obes Surg. 2024 Feb 24;34(4):1113–21. doi: 10.1007/s11695-023-07020-4 (PMC11026254; doi:10.1007/s11695-023-07020-4)
Supplement: Supplementary file 4 — Supplementary file4 (DOCX 41.7 KB) [file 11695_2023_7020_MOESM4_ESM.docx]

## Appendix C – BOSATS LRYGB – Observed items.

Relevant items of the BOSATS for a lineair stapled antecolic gastro-jejunostomy. [23]

|  | **Task / step.** | **1** | **2** | **3** | **4** | **5** | **N/A** |
| --- | --- | --- | --- | --- | --- | --- | --- |
|  | **Placement of liver retractor** | |  |  |  |  |  |
|  | **Elevate the transverse colon cephalad and identify ligament of Treitz** | Not performed |  | Performed in a traumatic fashion; ligament identified |  | Elevated smoothly and gently; good exposure of ligament |  |
|  | **Measure approximately 40-60cm of jejunum distal to the ligament of Treitz** | Length not measured |  | Measured, however individual measurements not of the same size; poor orientation |  | Measured methodologically; each measurement of the same size; correct orientation |  |
|  | **Confirm that this part of jejunum will reach the hiatus / gastric pouch** | Not confirmed |  | Confirmed briefly |  | Confirmed clearly and methodologically |  |
|  | **Divide jejunum** | Divided with tissue trauma and gross contamination |  | Bowel divided; jaws of stapler not perpendicular to bowel; possibility of mesenteric trauma; need for re-resection |  | Bowel divided at correct angle; good viability of ends; no trauma to mesentery |  |
| **Creation of the JJ anastomosis** | |  |  |  |  |  |  |
|  | **Preparation** |  |  |  |  |  |  |
|  | **Measure 75-150cm length of Roux-limb** | Length not measured |  | Measured, however individual measurements not of the same size; poor orientation; grabs onto mesentery |  | Measured methodologically; each measurement of the same size; correct orientation |  |
|  | **Bring distal end of BPL and position side to side to Roux limb** | Incorrect orientation of BPL to Roux limb |  | Correct orientation, although required multiple attempts with extra movements |  | BPL positioned correctly in relation to Roux limb; no extra movements; no tension |  |
|  | **Create enterotomies in BP and Roux limbs** | Poor relation between grasper and energy source; excessively large or small; penetration of posterior bowel wall |  | Appropriate size enterotomy not placed in antimesenteric location |  | Appropriately sized and placed enterotomies; no extra movements. Good relation of grasper and energy source |  |
|  | **Stapling** |  |  |  |  |  |  |
|  | **Insert the limbs of linear cutting stapler into the enterotomies in Roux and BPLs** | Unclear of how to insert the staple device. Drives staple with jaws blindly into BP and Roux limb |  | Inserts the stapler hesitation and lacks appreciation of the ideal angle for insertion |  | Inserts staple jaws with ease;controlled manner; correct angle |  |
|  | **Ensure both limbs are symmetrical and stapler on antimesenteric border** | Does not ensure limb symmetry and antimesenteric position before closure of jaws |  | Limbs either non-symmetrical or not on antimesenteric border before closure of jaws |  | Correctly ensures symmetry and anti-mesenteric position before closure of the jaws |  |
|  | **Fire stapler** | Uncontrolled fire with excessive pull on the bowel and widening of enterotomies |  | Controlled fire; some slippage of bowel from jaws |  | Smooth, controlled fire; no widening of enterotomies |  |
|  | **Close the created enterotomy with a simple running suture or a linear cutting stapler** | Poorly positioned stitch or stapler. Blindly placed sutures; traumatic use of needle drivers; skiving and poor control of needle; too much or too little tension; poor quality knot |  | Adequate stitch or stapler position. Adequate closure of enterotomy. Sutures placed at varying distances apart; gathering of bowel edges; additional reinforcement sutures required; stitch pulled out clumsily |  | Correct stitch or stapler position. Adequate size bites placed uniform distance apart; perpendicular to seromuscular layer; appropriate tension; adequate surgical knot; needle retrieved safely |  |
| **Dissection of the phreno-esophageal ligament (angle of His)** | | | |  |  |  |  |
|  | **Place patient in steep reverse Trendelenburg** | Patient not repositioned |  | Patient repositioned after prompting |  | Patient repositioned without delay |  |
|  | **Pull fundus of stomach down (exposure)** | Insufficient retraction; traumatic; insufficient exposure |  | Satisfactory retraction after some repositioning; suboptimal exposure |  | Appropriate retraction; optimal exposure |  |
|  | **Create a tunnel between the left crus of diaphragm and fundus of the stomach** | Incorrect location; associated trauma, bleeding |  | Correct location after multiple attempts; minimal tissue trauma, bleeding |  | Correct location; no difficulty or excessive tissue trauma |  |
|  | **Dissect angle of His close; keeping tension on fundus** | Dissection in incorrect plane insufficient or too much tension; bleeding |  | Dissection in correct plane;appropriate tension majority of the time; occasional tissue damage, bleeding |  | Dissection in correct plane; careful tension at all times; minimal tissue damage, bleeding |  |
| **Creation of the gastric pouch** | | |  |  |  |  |  |
|  | **Dissect along lesser curvature of stomach 3e8 cm from the gstroesophageal junction and keep close to stomach** | Incorrect plane; incorrect anatomic location; excessive tissue trauma; bleeding with need for suction |  | Correct plane developed with some difficulty; moderate tissue trauma; bleeding not requiring suction |  | Correct plane in correct anatomic location developed without difficulty or excessive tissue trauma, bleeding |  |
|  | **Create a posterior tunnel** | Dissection in incorrect plane; unnecessary force; bleeding requiring suction |  | Dissection in correct plane; occasional tissue damage; bleeding not requiring suction |  | Dissection in correct plane; careful handling of tissues, minimal tissue damage, bleeding |  |
|  | **Introduce and apply a linear cutting stapler transversely to the stomach** | Stapler applied in incorrect orientation; serosal damage to stomach |  | Stapler applied transversely after multiple repositioning attempts |  | Stapler applied transversely; no requirement for multiple repositioning attempts; no trauma to stomach wall |  |
|  | **Remove all tubes from the stomach before firing the stapler** | Not done |  | Done after delay; with prompting |  | Done without delay |  |
|  | **Fire stapler** | Uncontrolled fire with excessive pull on the stomach |  | Controlled fire; some slippage of stomach between jaws |  | Smooth, controlled fire |  |
|  | **Develop a posterior tunnel towards the angle of His** | Dissection in incorrect plane unnecessary force; bleeding requiring suction |  | Dissection in correct plane;occasional tissue damage; bleeding not requiring suction |  | Dissection in correct plane; careful handling of tissues, minimal tissue damage, bleeding |  |
|  | **Clean up posterior attachments (if present and prevent introduction of stapler) between the stomach and pancreas in the lesser sac** | Posterior attachments not cleared; excessive force, tissue trauma, bleeding requiring suction |  | Majority of attachments cleared up; moderate tissue trauma, bleeding not requiring suction |  | Attachments cleared; careful handling of tissues; minimal tissue trauma, bleeding |  |
|  | **Introduce and apply another linear cutting stapler to the stomach** | Stapler applied in an incorrect orientation; serosal damage to stomach |  | Stapler applied correctly; multiple repositioning attempts |  | Stapler applied correctly; no repositioning required; no trauma to stomach wall |  |
|  | **Fire stapler** | Uncontrolled fire with excessive pull on the stomach |  | Controlled fire; some slippage of stomach between jaws |  | Smooth, controlled fire |  |
|  | **Confirm complete transection of stomach** | Not confirmed |  | Confirmed briefly without adequate visualization |  | Methodical confirmation of complete transection |  |
| **Positioning of the Roux limb:** | | |  |  |  |  |  |
|  | **Antecolic, antegastric** |  |  |  |  |  |  |
|  | **If required, divide omentum to the transverse colon with an energy source** | Not performed when necessary; excessive tissue trauma; bleeding; injury to colon |  | Performed appropriately; moderate tissue trauma; bleeding |  | Performed correctly; minimal tissue trauma; bleeding |  |
|  | **Bring up Roux limb antecolic and antegastric** | Incorrect position; excessive tension or twisting of limb |  | Correct positioning; repositioning required; limb slips |  | Correct positioning; minimal tension or twisting |  |
| **Creation of the GJ anastomosis:** | | |  |  |  |  |  |
|  | **Linear stapler technique** |  |  |  |  |  |  |
|  | **Create a gastrotomy in the gastric pouch** | No entry into gastric lumen;poor relation between grasper and energy source; excessively large or small; penetration of posterior bowel wall; skiving; bleeding |  | Entry into gastric lumen; appropriate size; more than 1 attempt required |  | Entry into gastric lumen; appropriate size; no extra movements required |  |
|  | **Create an enterotomy in the Roux limb** | No entry into bowel lumen; poor relation between grasper and energy source; excessively large or small; penetration of posterior bowel wall |  | Appropriate size and entry into bowel lumen; not placed in antimesenteric location |  | Appropriate size and placement of enterotomy; good relation of grasper and energy source; no extra movements required |  |
|  | **Introduce one limb of linear cutting stapler into gastric pouch and the other into Roux limb** | Unclear of how to insert the staple device; drives staple jaws blindly into the enterotomies |  | Inserts the stapler, but lacks appreciation of the ideal angle for insertion |  | Inserts staple jaws with ease; controlled manner; correct angle |  |
|  | **Ensure both limbs are symmetrical before firing the stapler** | Does not ensure symmetry, antimesenteric location of stapler before closing of jaws |  | Limbs either nonsymmetrical or not on antimesenteric border before closure of jaws |  | Correct symmetry and antimesenteric position before closure of jaws |  |
|  | **Fire stapler** | Uncontrolled fire with excessive pull on the bowel and widening of enterotomies |  | Controlled fire; some slippage of bowel from jaws |  | Smooth, controlled fire; no widening of enterotomies |  |
|  | **Suture closed the enterotomy** | Poorly positioned stitch; blindly placed sutures; traumatic use of needle drivers; skiving and poor control of needle; too much or too little tension; poor quality knot |  | Adequate stitch position; adequate closure of enterotomy. Sutures placed at varying distances apart; gathering of bowel edges; additional reinforcement sutures required; stitch pulled out clumsily. |  | Correct stitch position; adequate size bites placed uniform distance apart; perpendicular to seromuscular layer; appropriate tension; adequate surgical knot; needle retrieved safely |  |
|  | **Ensure cinching of suture between bites** | Not done |  | Done inconsistently |  | Done at all times |  |
| **Testing of GJ anastomosis:** | |  |  |  |  |  |  |
|  | **Blue dye technique** |  |  |  |  |  |  |
|  | **Clamp Roux limb distal to GJ anastomosis** | Not clamped; clamped with excessive tissue trauma, traction |  | Clamped correctly; mild tissue trauma or traction; some clamping of mesentery |  | Clamped correctly; minimal tissue trauma or traction |  |
|  | **Fill gastric pouch with 60-120 mL of methylene blue dye** | Not performed; insufficient amount of dye to distend the gastric pouch |  | Performed correctly with some prompting; moderate distension of gastric pouch |  | Performed correctly; adequate distension of gastric pouch |  |
|  | **Suction at left and right corners of anastomosis looking for dye leak** | Not performed |  | Performed hastily |  | Performed carefully and thoroughly |  |
|  | **Request for fluid to be sucked back from gastric pouch** | Not performed |  | Performed after some delay |  | Performed quickly, without delay |  |
|  | **Air insufflation test** |  |  |  |  |  |  |
|  | **Introduce an endoscope/ orogastric tube into gastric pouch** | Not performed; excessive tissue manipulation/trauma |  | Performed correctly; prompting required; minimal tissue trauma |  | Performed correctly; without delay; minimal tissue trauma |  |
|  | **Instill saline into peritoneal cavity and submerge the GJ in saline** | Not performed |  | Performed after some delay |  | Performed quickly, without delay |  |
|  | **Occlude Roux limb distal to GJ anastomosis** | Not clamped; clamped with excessive tissue trauma, traction |  | Clamped correctly; mild tissue trauma or traction; some clamping of mesentery |  | Clamped correctly; minimal tissue trauma or traction |  |
|  | **Insufflate gastric pouch with air** | Not performed; insufficient amount of air to distend the gastric pouch |  | Performed correctly with some prompting; moderate distension of gastric pouch |  | Performed correctly; adequate distension of gastric pouch |  |
|  | **Check for air leak** | Not performed |  | Performed hastily |  | Performed carefully and thoroughly |  |
|  | **Suction saline from peritoneal cavity** | Not performed |  | Performed after some delay |  | Performed quickly, without delay |  |
