# Supplementary material for: A New Procedure-Based Assessment of Operative Skills in Gastric Bypass Surgery, Evaluated by Video Fragment Rating
Source: Obes Surg. 2024 Feb 24;34(4):1113–21. doi: 10.1007/s11695-023-07020-4 (PMC11026254; doi:10.1007/s11695-023-07020-4)

**Appendix D – Study Website**

The study website showing three video links including the assessments, accompanied by the two additional questionnaires – surgical experience and assessment preference.


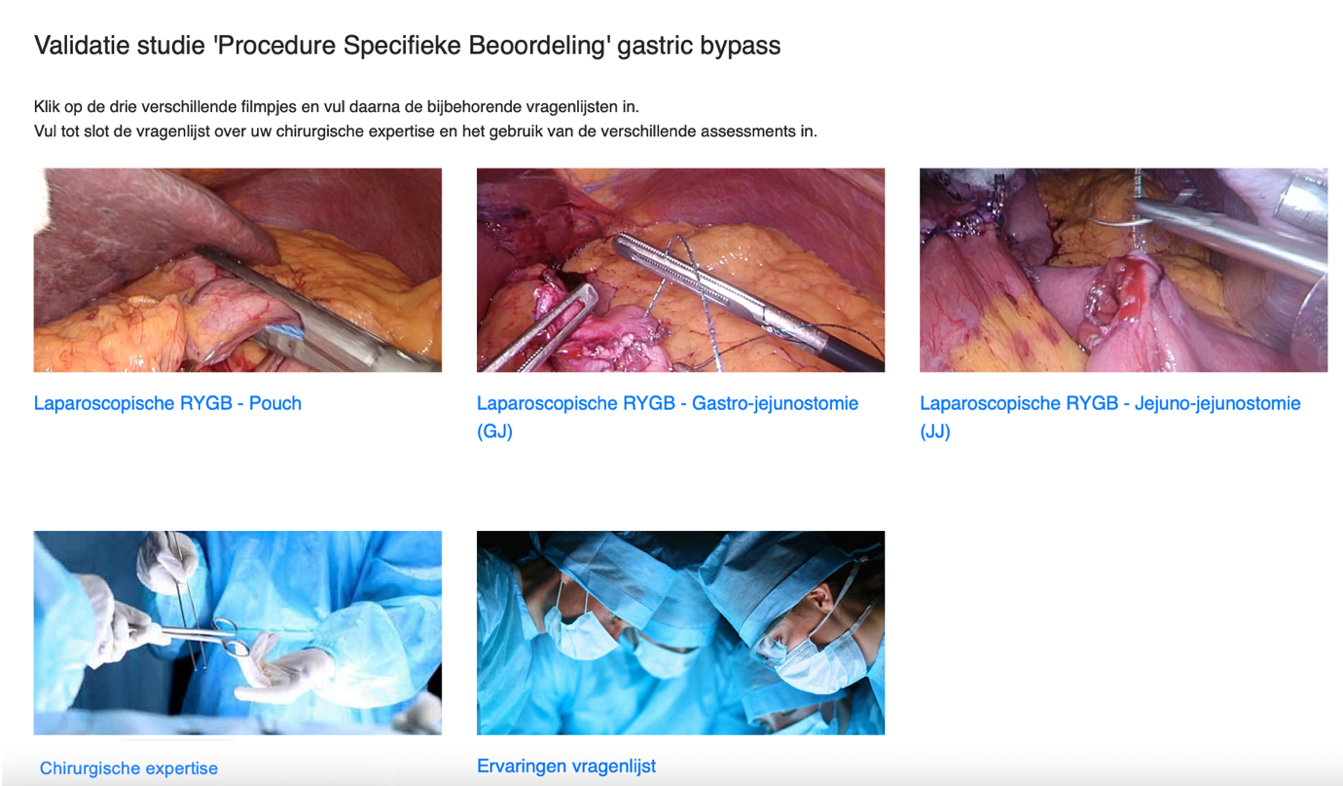

Supplement: Supplementary file 5 — Supplementary file5 (DOCX 1.34 MB) [file 11695_2023_7020_MOESM5_ESM.docx]
