# Supplementary material for: A New Procedure-Based Assessment of Operative Skills in Gastric Bypass Surgery, Evaluated by Video Fragment Rating
Source: Obes Surg. 2024 Feb 24;34(4):1113–21. doi: 10.1007/s11695-023-07020-4 (PMC11026254; doi:10.1007/s11695-023-07020-4)
Supplement: Supplementary file 6 — Supplementary file6 (DOCX 14.2 KB) [file 11695_2023_7020_MOESM6_ESM.docx]

## **Appendix E – Assessment** **evaluation**

All experts answered this evaluation for all three assessments. In case of the OSATS the question ‘should also be made for other laparoscopic procedures’; was omitted, as this is a GRS.

|  | **Strongly disagree** | **Disagree** | **Neutral** | **Agree** | **Strongly agree** |
| --- | --- | --- | --- | --- | --- |
| Gives a correct judgment about the competence to perform a specific procedure | 1 | 2 | 3 | 4 | 5 |
| Leads to an unnecessary administrative burden | 1 | 2 | 3 | 4 | 5 |
| Should be used in clinical practice | 1 | 2 | 3 | 4 | 5 |
| Helps in the acquirement of procedural knowledge and skills | 1 | 2 | 3 | 4 | 5 |
| Should also be made for other laparoscopic procedures | 1 | 2 | 3 | 4 | 5 |
|  | **Subjective** | **Between neutral and subjective** | **Neutral** | **Between neutral and objective** | **Objective** |
| Is objective or subjective | 1 | 2 | 3 | 4 | 5 |
